# Melatonin Ameliorates decaBDE-Induced Autism-Relevant Behaviors Through Promoting SIRT1/SIRT3/FOXO3a-Dependent Mitochondrial Quality Control

**DOI:** 10.3390/antiox15030405

**Published:** 2026-03-23

**Authors:** Lu Gao, Jinghua Shen, Jingjing Gao, Tian Li, Dongying Yan, Xinning Zeng, Jia Meng, Hong Li, Dawei Chen, Jie Wu

**Affiliations:** 1 Department of Occupational and Environmental Health, School of Public Health, Jinzhou Medical University, Jinzhou 121001, China; gaol@stu.jzmu.edu.cn (L.G.); shenjh@stu.jzmu.edu.cn (J.S.);; 2 School of Public Health, Jinzhou Medical University, Jinzhou 121001, China; 3 NHC Key Laboratory of Food Safety Risk Assessment, Chinese Academy of Medical Science Research Unit (No. 2019RU014), China National Center for Food Safety Risk Assessment, Beijing 100021, China

**Keywords:** autism spectrum disorder, decabromodiphenyl ether, melatonin, mitochondrial quality control, sirtuins

## Abstract

The etiology of autism spectrum disorder (ASD) implicates genetic predispositions and environmental chemicals, such as polybrominated diphenyl ethers (PBDEs). We aimed to identify whether mitochondrial quality control (MQC) was involved in ASD-relevant behavioral changes induced by decabromodiphenyl ether (deca-BDE, BDE-209) and the alleviation by melatonin. Pregnant rats exposed to BDE-209 (50 mg/kg i.g.) were administrated melatonin through drinking water (0.2 mg/mL) during gestation and lactation. Behavioral assessments integrated open-field test, three-chamber social test, and Morris water maze; mitochondrial detections took transmission electron microscopy, immunofluorescence, and homeostasis together; hippocampal molecular network was identified through transcriptomics profiles, combining dendritic morphology analysis after Golgi-Cox staining. Melatonin supplementation attenuated BDE-209-reduced social and cognitive ability, accompanied by improvements in hippocampal synaptic plasticity (dendritic spines, PSD95, SNAP25). Mitochondrial dysfunctions, shown as decreases in complex IV activity, ATP content, and mtDNA copies, plus redox imbalance (ROS/SOD2) and resultant mitochondrial membrane potential disruption and apoptosis, together with fusion/fission dynamic (MFN2/DRP1), biogenesis (SIRT1-PGC1α-TFAM), and mitophagy (SIRT3-FOXO3-PINK1) suppression, were reversed by melatonin partially through SIRT1 (Sirtuin-1)-dependent pathways, as these protections were abolished by inhibitor EX527. This study highlighted the SIRT1–SIRT3 axis in MQC and behavioral effects, providing novel intervention for PBDEs’ neurodevelopmental impairment.

## 1. Introduction

Autism spectrum disorder (ASD) is a neurodevelopmental disorder characterized by social communication impairments and restricted, repetitive behaviors [1]. The estimated global prevalence of ASD has been increasing to approximately 100/10,000, and the median percentage of individuals with co-occurring intellectual disability among autism is 33.0% [2]. In terms of etiology, both genetic heritability and environmental chemicals are decisive factors [1,3]. Polybrominated diphenyl ethers (PBDEs), such as brominated flame retardants (BFRs), were widely used in various household products and materials before phase-out by the Stockholm Convention [4]. Children’s body burden of PBDEs has been reported to be significantly higher (approximately four to six folds) than adults, partially attributed to breast milk [5]. In addition, exposure to decabromodiphenyl ether (deca-BDE, BDE-209) in manufacturing and e-waste recycling has raised occupational health hazards recently [6,7]. PBDEs are developmental neurotoxicants, and both pre- and postnatal exposure lead to cognitive behavioral deficits [8]. Moreover, maternal transfer of a commercial mixture of PBDEs, DE-71, resulted in ASD-relevant behavioral and neurochemical abnormality in female offspring [3,9], while the underlying mechanisms remain obscure. Mitochondria are crucial for energy production via oxidative phosphorylation (OXPHOS), which generates adenosine triphosphate (ATP) [10]. Energy-intensive cells such as neurons are particularly vulnerable to mitochondrial dysfunction; as expected, abnormal mitochondrial function is prevalent in individuals with ASD, where about 8~47% demonstrate corresponding biomarkers [11]. The 16.6 kb human mitochondrial genome (aka mitochondrial DNA), located within the mitochondrial matrix, encodes essential proteins for mitochondrial function [12]. Decreased mtDNA content is associated with ASD [13], as Mendelian randomization revealed a 27% decrease in the risk of ASD per standard deviation increase in genetically determined blood mtDNA copy number (odds ratio, OR = 0.73) [14]. Furthermore, exposure to BDE-209 and its substitute decabromodiphenyl ethane (DBDPE) was negatively correlated to mtDNA copy number, indicating that mitochondrial dysfunction was involved [15].

To combat environmental stressors, a sophisticated mitochondrial quality control (MQC) mechanism has evolved, encompassing key processes: mitochondrial biogenesis, mitochondrial dynamics, and mitophagy [16]. The sirtuin (SIRT) family refers to seven conserved nicotinamide adenine dinucleotide (NAD)-dependent histone deacetylases (SIRT1–SIRT7); notably, SIRT1–SIRT3 exert neuroprotective effects by regulating synaptic and mitochondrial function [17]. SIRT1 upregulates the activity of peroxisome proliferator activated receptor (PPAR) γ coactivator 1α (PGC 1α) by promoting its deacetylation, thereby enhancing mitochondrial quality and biogenesis via facilitating the transcription of mitochondrial transcription factor A (TFAM) and the replication of mtDNA [18]. Furthermore, PGC 1α regulates the formation and maintenance of dendritic spines in developing and adult hippocampal neurons [19]. SIRT3 modulates mitochondrial metabolism and cooperates with SIRT1 to increase the life span of experimental animals [20], such as by deacetylating Forkhead box O-3 α (FOXO3α), which has been shown to trigger antioxidant responses via modulation of SOD2 and CAT gene expression [21], mediate mitochondrial fusion/fission proteins at transcription (mitofusin 2, Mfn2) and post-translation (Drp1 dephosphorylation) [22], and induce the initiation of the PTEN-induced putative kinase 1 (PINK1)-Parkin mitophagy pathway to inhibit cell death [23].

Oxidative stress and apoptosis, as well as downregulation of synaptic proteins, are abundantly involved in PBDEs’ neurotoxicity [24,25], the exposure of which causes ATP depletion, mitochondrial membrane potential (MMP) loss and mitochondrial dysfunction. Mechanistically, BDE-47 suppressed PGC-1α/NRF1/TFAM signaling pathways and mtDNA encoding protein synthesis [26], and SIRT3/FOXO3α/PINK1 pathway inhibition was also induced by BDE-209 [24]. Furthermore, numerous reports have shown that the activity of SIRT1 is enhanced in neurological disorders after treatment with melatonin (N-acetyl-5-methoxytryptamine), a pleiotropic neurohormone, including autism rats [27,28]. We also found that downregulation of SIRT1 in both hippocampal microglia and neurons of offspring rats after perinatal BDE-209 exposure was reversed by melatonin [29,30]. Therefore, we aimed to further identify whether mitochondrial homeostasis together with biogenesis and mitophagy were involved in the developmental neurotoxicity of BDE-209, and the alleviation of ASD-relevant behavioral changes from melatonin.

## 2. Materials and Methods

### 2.1. Chemicals and Reagents

Decabromodiphenyl ether (deca-BDE or BDE-209, 98%, CAS#1163-19-5) was obtained from J & K Chemical Technology (Beijing, China); sodium valproate (VPA, powder, 98%, CAS#1069-66-5) and melatonin (MT, powder, ≥98%, CAS#73-31-4) were from Sigma-Aldrich Trading Co., Ltd. (Shanghai, China); Sirt1/Sir2 inhibitor Selisistat (EX527, 99.85%, CAS#49843-98-3) was from MedChemExpress (Shanghai, China). Primary antibodies used for Western blotting were as follows: GAPDH (60004-1-Ig) and PSD95 (20665-1-AP) were from Proteintech (Wuhan, China); SNAP25 (sc-73044), cytochrome c (sc-13156), caspase-3 (sc-56053), COX4 (sc-517553), DRP1 (sc-271583), FIS1 (sc-376447), MFN2 (sc-100560), OPA1 (sc-393296), NRF1 (sc-28379), TFAM (sc-166965), SOD2 (sc-133134), PINK1 (sc-517353), and PRKN (sc-32282) were from Santa Cruz (Dallas, TX, USA); Acetylated-Lysine Antibody (#9441, CST, Danvers, MA, USA); SIRT1 (13161-1-AP), SIRT3 (10099-1-AP), PGC1α (66369-1-Ig), and FOXO3a (66428-1-Ig) were from Proteintech (Wuhan, China).

### 2.2. Animals and Treatment

Ten-week-old Sprague-Dawley rats (weighing 280 g ± 30 g) were obtained from Changsheng Biotechnology (Liaoning, China, license number: SYXK 2019-0007). All the animals were housed at standard conditions of temperature (22 ± 2 °C) and relative humidity (50 ± 5%) under a 12/12 h light/dark cycle, with access to food and water ad libitum. Female and male rats were mated at a 2:1 ratio after 1-week acclimation, and the day of the vaginal plug appearance was recorded as gestation day 0. Pregnant rats were randomly divided into five groups: control (CON), BDE-209 (BDE), MT + BDE-209 (MTB), MT + BDE-209 + EX527 (MTB + EX), and autism model (VPA), 5~6 dams/litters each group. MT (0.2 mg/mL, drinking water in light-protected bottles, approximately 20 mg/kg daily) and BDE-209 (50 mg/kg, i.g., 10:00 a.m. to 11:00 a.m.) were administrated during pregnancy and lactation as previously described [27,31], and VPA was administrated at gestational day 12.5 (500 mg/kg, i.p.) [27]. EX527 (5 mg/kg, i.p.) was administrated to pup rats every 2 days [32] for 2 weeks from postnatal day (PND) 14 to 28. Control group rats received corn oil or saline instead. A dose of 50 mg/kg is about ten-fold the environmental equally calculated dose, depending on estimated daily intake (18.7 μg/kg/day, closed to the benchmark dose for a 10% neurodevelopmental toxicity effects—17 μg/kg/day [33]) in the deca-BDE-manufacturing plant, as recently reported [7], under the uncertainty factor of 300 used for RfD. The body weight was recorded every two days to adjust dosage volume, and abnormal maternal behavior or pup growth was observed under above treatment. The Animal Ethics Committee of Jinzhou Medical University (No. 240123) approved the present experimental program, which complied with the National Institutes of Health Guide for the Care and Use of Laboratory Animals.

### 2.3. Behaviors

According to previous studies, environmental endocrine-disrupting chemicals, including polybrominated compounds, caused changes in anxiety-like and social behaviors only in females, while stress was predominantly in male rats [34]; developmental DE-71-exposed (0.1 or 0.4 mg/kg/day) adult F1 female offspring displayed deficient short- and long-term novel recognition memory and persistent autism-like behaviors [3]. Therefore, in this study, we focused on behavioral and mechanistical measurements only in female adolescent rats after BDE-209 exposure. During behavioral assessments (PND 35 to 50, from 09:00 a.m. to 04:00 p.m.), randomization and double-blinding protocols were strictly adhered to, with all testing procedures conducted by personnel unaware of group allocations.

#### 2.3.1. Open-Field Test (OFT)

In the open-field test, rats were placed in the apparatus (100 cm × 100 cm × 45 cm) and allowed to freely explore for 10 min; their movements were recorded by tracking master V3.0 software (Zhongshi Technology, Beijing, China). After each test session, the box was thoroughly cleaned with 70% ethanol to maintain a standardized testing environment. The key parameters related to locomotor activity were quantified, including total distance traveled and entries and time spent in the center zone.

#### 2.3.2. Three-Chamber Social Test

This test was conducted to evaluate social behavior of rats. The apparatus used was a plexiglass box with dimensions of 62 cm × 42 cm × 24 cm, divided into a central chamber and two side chambers. Each side chamber provided a cylindrical cage with a diameter of 8 cm. A video recording system with VisuTrack software (Xinruan Technology, Shanghai, China) continuously tracked movement and behavior throughout the test. Prior to formal tests, animals underwent 10 min habituation of all three chambers, without showing positional preferences. The first part was sociability test, with a novel object (empty cage (‘E’)) and a novel rat (an age- and sex-matched Stranger 1 in cage: ‘S1’) introduced into each side chamber, respectively, and the subject rats were allowed to explore freely for 10 min. The positions of the novel object and the stranger rat were randomized to avoid side preference. An hour later, the second part tested social novelty preference via another 10 min exploration, with a second novel unfamiliar rat (Stranger 2: ‘S2’) put into the previous empty cage. The time spent in each chamber and on sniffing the rats was recorded, while preference index for sociability was defined as 100 × (S1 − E)/(S1 + E), and the preference index for social novelty was defined as 100 × (S2 − S1)/(S2 + S1) [35].

#### 2.3.3. Morris Water Maze (MWM)

This test was performed to evaluate spatial learning and memory of rats. During 4 days of training, a 10 cm diameter platform was submerged 1 cm below the water surface in the middle of the target quadrant. Subject rats underwent four trials daily, initiated from each quadrant in a pseudorandom sequence, to locate the platform. On the 6th day, a probe trial was conducted after removing the platform. The rats were released into the water from the quadrant opposite the previous platform location and swam for 60 s. Their swimming paths were tracked using a digital camera and the ANY-maze tracking System (Stoelting, Wood Dale, IL, USA). The number of times they crossed the target quadrant and the time spent there were recorded to assess spatial memory.

### 2.4. Transmission Electron Microscopy (TEM)

Rats were anaesthetized with 1% pentobarbital sodium (30 mg/kg, i.p.) and perfused with phosphate buffer (PBS). The hippocampus was obtained and sectioned into 1 × 1 × 1 mm^3^ pieces quickly, then immersed in 2.5% glutaraldehyde overnight at 4 °C. Following dehydration in ethanol series, the samples were embedded in resin. Subsequently, ultrathin sections measuring 70 nm thickness were prepared and embedded on a 200 mesh copper grid. The grid was then stained with 4% uranyl acetate for 80 min and 0.4% lead citrate for 3 min [35]. The ultrastructure of neuronal mitochondria was examined using a transmission electron microscope (Hitachi 7650, Toyko, Japan).

### 2.5. Golgi-Cox Staining

Hippocampal CA1 dendritic morphology was demonstrated utilizing a Rapid GolgiStainTM Kit (FD NeuroTechnologies, Ellicott City, MD, USA) as previously described [30]. Briefly, rats’ brains were immersed in impregnation solution (A + B) for 2 weeks at room temperature (RT) in the dark, then transferred into Solution C for at least 72 h. Coronal sections (100 μm thick, 5~6 sections each area per rat) were prepared at −22 °C using a Leica cryostat (CM1950, Wetzlar, Germany) after being snap-frozen in dry ice and mounted on gelatin-coated slides. After drying naturally (24 h/RT/dark), the slices were stained in Solution D + E/Milli-Q water for 10 min, dehydrated with graded ethanol series (50%, 75%, 95%, and twice 100%, 4 min each), cleared in xylenes, and finally mounted with Permount™ Mounting Medium (Solarbio, Beijing, China). Pyramidal neuron arborization and spines were imaged via Olympus BX53F microscope (Tokyo, Japan) and analyzed via Fiji ImageJ v1.8.0 (NIH, Bethesda, MD, USA).

### 2.6. Double-Labeling Immunofluorescence

Coronal sections (10 μm thick) including the hippocampus were prepared using a cryostat microtome, following transcardial perfusion and further fixation with paraformaldehyde and sinking in sucrose solution. During immunofluorescence staining, sections were blocked with 5% goat serum (Solarbio, Beijing, China) for 1 h at RT, then incubated with specific primary antibodies at 4 °C overnight: mouse anti-TOM20 (1:50, sc-17764, Santa Cruz, Dallas, TX, USA), rabbit anti-DRP1 (1:100, A20125) and PINK1 (1:100, A58164; Nature Biosciences, Hangzhou, China). After washing with PBS, the sections were incubated with CoraLite488/594-conjugated goat anti-rabbit or mouse IgG (1:200, Proteintech, Wuhan, China) for 1 h at RT under dark. After washing, the sections were mounted with DAPI-containing anti-quench medium (Beyotime, Nanjing, China). Fluorescence images were taken with a Leica M165FC microscope (Wetzlar, Germany) and analyzed via ImageJ software.

### 2.7. Mitochondrial Homeostasis Detection

#### 2.7.1. Mitochondrial DNA (mtDNA) Content

Total DNA from the rat hippocampus was extracted following the manufacturer’s protocol (Takara #9765, Dalian, China). Briefly, hippocampal samples were minced, followed by enzymatic digestion using Buffer GL, proteinase K, and RNase A at 56 °C for 3 h. The lysate was clarified through centrifugation at 12,000 rpm for 2 min and ethanol-precipitated with Buffer GB before undergoing spin-column purification. Subsequent column washes with Buffer WA and WB at 12,000 rpm for 1 min each were performed prior to a final desiccation centrifugation lasting 2 min. DNA elution was carried out at room temperature for 5 min of equilibration, and purified DNA was quantified using micro-spectrophotometer (NanoDrop, Thermo Fisher, Waltham, MA, USA). Finally, mtDNA and nuclear DNA were quantified by qPCR Kit (Takara # RR820A, Dalian, China), with primers shown in Table 1.

#### 2.7.2. COXIV Activity Assays

The mitochondrial respiratory chain complex IV levels in the hippocampus were quantified using an ELISA kit (Mlbio, Shanghai, China). Freshly dissected hippocampus was minced and homogenized in PBS. The homogenate was centrifuged at 3000× *g* for 20 min, and the supernatant was collected for assay. Following the manufacturer’s instruction, samples and standards were added to the wells of the microplate; after incubation and termination, OD (optical density) values were measured using a microplate reader (DeTie HBS-1096A, Nanjing, China) at 450 nm.

#### 2.7.3. Mitochondria Isolation and ATP Content

Mitochondria extraction from the hippocampus was performed following the manufacturer’s directions (Beyotime #C3606, Shanghai, China). The procedure involved mincing fresh tissues and homogenization. The homogenate underwent centrifugation at 1000× *g* for 5 min to isolate purer mitochondria. The resulting supernatant was then subjected to further centrifugation at 11,000× *g* for 10 min to collect the mitochondrial pellet.

ATP levels in hippocampal mitochondria were determined by an ATP assay kit (Beyotime #S0026, Shanghai, China). Mitochondrial pellets were homogenized in ATP lysis buffer and centrifuged at 12,000× *g* for 5 min to collect the supernatant. The ATP detection working solution was then added to the assay wells for 5 min to deplete background. Finally, samples or standards were added and mixed thoroughly, and RLU values were measured using a luminometer (Feyond-300, ALLSHENG, Hangzhou, China). ATP concentrations were calculated based on the standard curve and normalized by protein concentration [35].

#### 2.7.4. SOD Activity Assays

Superoxide dismutase (SOD) activity was quantified using a total SOD assay kit (Beyotime #S0101, Shanghai, China). Fresh hippocampal homogenate was centrifuged at 12,000× *g* for 5 min, and the supernatant was collected for analysis. The reaction working solution was prepared according to the manufacturer’s protocol. Following incubation at 37 °C for 30 min, absorbance was measured using a microplate reader at 450 nm.

#### 2.7.5. Determination of Reactive Oxygen Species (ROS)

Hippocampal cell suspension was obtained as described previously [29]. Under deep anesthetization, rats’ hippocampus were isolated and minced on ice, then digested in 0.25% trypsin (Genview, Beijing, China) at 37 °C for 15 min with gentle shaking. After termination by 20% fetal bovine serum (Solarbio, Beijing, China), the suspensions were filtered through a 70 μm nylon filter and centrifuged at 1000× *g* for 5 min. The cell pellets were subsequently incubated in DCFH-DA probe solution (Beyotime #S0033S, Shanghai, China) at 37 °C for 20 min. After washing and resuspension in PBS, fluorescence intensity of DCF was assessed by flow cytometry to detect intracellular ROS levels.

#### 2.7.6. JC1 for Mitochondrial Membrane Potential

JC-1 (Beyotime #C2006, Shanghai, China) serves as a suitable fluorescent probe for assessing mitochondrial membrane potential (Δψm). Neural cells obtained from fresh hippocampal tissues were incubated with JC-1 staining solution (1:200, 0.5 mL/1 × 10^6^ cells) at 37 °C for 20 min, centrifuged at 600× *g* for 3~4 min, washed twice and finally suspended in staining buffer. Fluorescence of JC-1 monomers (green) and aggregates (red) was assessed by flow cytometry.

### 2.8. Apoptosis

The Annexin V-FITC/PI apoptosis detection kit (KeyGen BioTech, Nanjing, China) was used. Hippocampal neural cells were collected and resuspended with Binding Buffer, including 5 μL Annexin V-FITC and 5 μL propidium iodide (PI), and incubation for 15 min at RT. After being washed and suspended in Binding Buffer, cells were acquired on a flow cytometer (FACSCelesta, BD Bioscience, Franklin Lakes, NJ, USA), and data were analyzed with FlowJo V10 software (BD, Ashland, OR, USA).

### 2.9. Transcriptomic Sequencing of RNA

Hippocampus extracted from rats’ brains was quickly frozen within liquid nitrogen then stored at −80 °C. To delineate molecular responses to BDE-209 or VPA exposure and melatonin remediation, mRNA sequencing was performed by Sangon Biotech Co., Ltd. (Shanghai, China). Differentially expressed genes (DEGs: log_2_FC > 1, FDR < 0.001) were clustered, and functional and pathway enrichment were performed via clusterProfiler utilizing Gene Ontology (GO) and Kyoto Encyclopedia of Genes and Genomes (KEGG) databases. Protein–protein interaction (PPI) networks were constructed via STRING 12.0 and visualized via Cytoscape 1.0.

### 2.10. RNA Extraction and Quantitative Real-Time PCR (qPCR)

Total RNA was extracted from hippocampus tissue using RNAiso Plus (Takara, Dalian, China) according to the manufacturer’s protocol. After being reverse-transcribed into cDNA with the PrimeScriptTM RT reagent Kit (Takara #RR047A, Dalian, China), qPCR was carried out on an Applied Biosystems™ Real-Time PCR System (Thermo Scientific, Waltham, MA, USA) using the TB Green Premix Ex TaqTM II Kit (Takara #RR820A, Dalian, China). The primer sequences used are presented in Table 1. The qPCR program consisted of an initial cycle at 95 °C for 30 s, followed by 40 cycles of 95 °C for 3 s and 60 °C for 30 s, and then a melt curve stage. The relative mRNA levels of the target genes were calculated by the 2^−ΔΔCt^ method normalized with *Gapdh*.

### 2.11. Co-Immunoprecipitation (Co-IP)

Total protein from hippocampus were extracted using NP-40 lysis buffer (Beyotime, Shanghai, China) and the interaction of SIRT1/PGC-1α, SIRT3/FoxO3a, and Acetylated-PGC-1α and FoxO3a were detected using the BeaverBeadsTM Protein A/G Immunoprecipitation Kit (Beaver #22202, Suzhou, China). Magnetic beads were bound with 1 μg antibody (rabbit anti-SIRT1, rabbit anti-SIRT3, mouse anti-PGC-1α, or mouse anti-FoxO3a) for 60 min at RT. After washed with Binding Buffer, the magnetic beads were interacted with hippocampal lysates on a rotator at 4 °C overnight. Following washing, the magnetic beads were transferred into a new Eppendorf tube. The antigen–antibody complexes were eluted using 1× SDS-PAGE loading buffer (Beyotime, Shanghai, China) and heated at 95 °C for 5 min. Finally, antibody-combined proteins were analyzed by Western blotting.

### 2.12. Protein Samples Extraction and Western Blotting

The Western blot assay was carried out following standard procedures. Briefly, total proteins and nuclear proteins from hippocampal tissues were extracted using Radio Immunoprecipitation Assay Lysis Buffer (RIPA) and an Extraction Kit (#KGB5302, KeyGen BioTech, Nanjing, China), respectively. Mitochondrial protein extraction was performed according to the manufacturer’s instructions (Beyotime #C3606, Shanghai, China). Protein samples were subjected to sodium dodecyl sulfate-polyacrylamide gel electrophoresis (SDS-PAGE) at 80~120 V, then transferred onto polyvinylidene difluoride (PVDF) membranes (100 V or 250 A, 50~60 min). The membranes were blocked with 5% non-fat dry milk dissolved in 1× TBST (Tris-buffered saline containing Tween), incubated with primary antibodies overnight at 4 °C, washed in 1× TBST, and incubated with an HRP-conjugated secondary antibody (1:5000, Abclonal, Wuhan, China) for 1 h at RT. Finally, the membranes were exposed using an ECL substrate (Beyotime, Shanghai, China) on a Chemiluminescence System (General Electric, Boston, MA, USA). The optical density of bands was quantified using ImageJ software, normalized to GAPDH or PCNA.

### 2.13. Statistical Analysis

Statistical analyses were conducted using SPSS (v26.0, IBM, Chicago, IL, USA) with graphical visualization via GraphPad Prism 8.0 (GraphPad Software Inc, San Diego, CA, USA), R (v4.4.1), and SRplot (https://www.bioinformatics.com.cn/) [36]. For comparison among groups, one-way analysis of variance (ANOVA) with post hoc Tukey’s test (variance homogeneity-dependent) or Tamhane’s T2 test was employed. Repeated-measures two-way ANOVA addressed navigation test of water maze. Data were represented as mean ± standard deviation (SD). Differences were regarded as statistically significant when *p* < 0.05.

## 3. Results

### 3.1. Maternal Melatonin Supplementation Mitigates Autism-Relevant Behaviors in decaBDE-Exposed Female Rats

Prenatal VPA exposure has been suggested as an experimental animal model for autism-like behaviors, characterized by social deficits. In addition, the individuals with ASD have higher rates of co-occurring anxiety (11% vs. 5%) and intellectual disability (23% vs. 0.7%) than typically developing children [1]. Therefore, three-chamber interaction, open-field test and Morris water maze were programmed to evaluate autism-relevant behavioral phenotype induced by developmental BDE-209 exposure, utilizing prenatal VPA-exposed model as a positive control.

**Figure 1:**
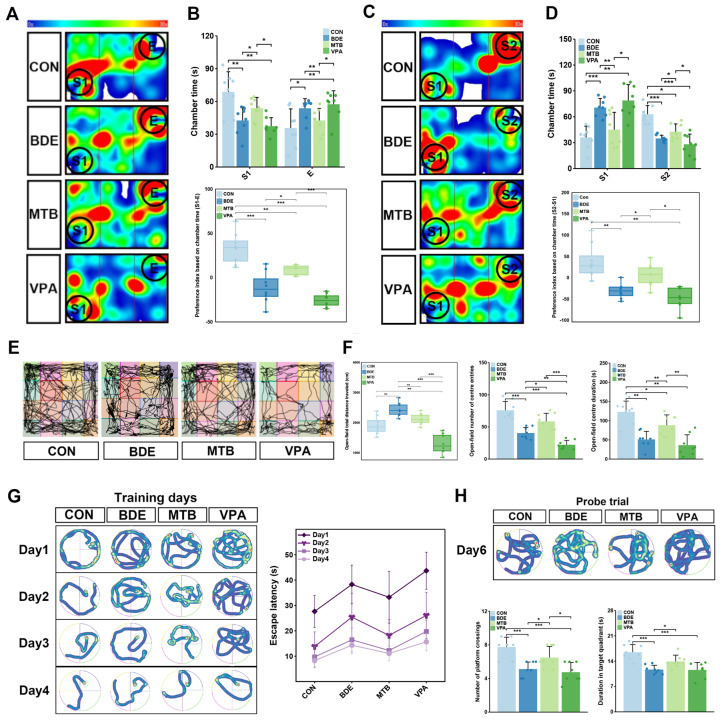
Maternal melatonin supplementation mitigates autism-relevant behaviors in decaBDE-exposed female rats. (**A**) Representative heatmaps depicting rat movement patterns during sociability test (E: empty cage; S1: stranger 1). (**B**) Time duration in S1 or empty cage with corresponding preference index. (**C**) Representative heatmaps depicting rat trajectories during social novelty preference test (S1: Stranger 1; S2: Stranger 2). (**D**) Comparative chamber occupancy duration between S1 and S2 and resulting social preference index. (**E**) Movement trajectory. (**F**) Total distance traveled, time spending and frequency entering centra area during open-field test (OFT). (**G**) Navigation heatmaps during 4-day acquisition phase and learning curve analysis showing daily escape latency in Morris water maze. (**H**) Trajectory heatmap during spatial probe trial (Day 5 post-training), with target quadrant duration and platform crossings. All data represent 8 biologically independent replicates, presented as mean ± SD. * *p* < 0.05, ** *p* < 0.01, *** *p* < 0.001.

In the three-chamber paradigm (Figure 1A–D), BDE (S1-E = −11.15) and VPA (S1-E = −25.58) groups exhibited reduced sociability indices compared to controls (S1-E = 33.07 vs. BDE, *p* = 0.0002; vs. VPA, *p* = 5.44 × 10^−7^), with less time in stranger-containing chambers (S2-S1: BDE = −31.33 vs. CON, *p* = 0.0077; VPA = −47.51 vs. CON, *p* = 0.0016), while MT intervention partially restored sociability and social novelty preference (S1-E: MTB = 8.75 vs. BDE, *p* = 0.0093; S2-S1: MTB = 6.12 vs. BDE, *p* = 0.0259).

Open-field assessments (Figure 1E,F) demonstrated hypoactivity in VPA model rats (total distance: CON = 1909.24 ± 296.68 cm, VPA = 1218.14 ± 321.53 cm, *p* = 2.6 × 10^−5^), while slight hyperactivity occurred in BDE-209-exposed female rats (2263.66 ± 261.48 cm, no significance vs. CON or MTB), accompanied by suppressed central zone exploration (entries: CON = 75.88 ± 13.59; BDE = 40.50 ± 8.86 vs. CON, *p* = 0.0008; VPA = 22.25 ± 682 vs. CON, *p* = 0.0007; duration: CON = 122.86 ± 28.27 s; BDE = 51.19 ± 21.23 vs. CON, *p* = 0.0004; VPA = 35.00 ± 24.75 vs. CON, *p* = 0.0005). MT administration improved enhanced central exploration frequency and duration (MTB: entries = 58.69 ± 12.54 s vs. BDE, *p* = 0.0157; duration = 88.45 ± 26.65 vs. BDE, *p* = 0.008).

Morris water maze acquisition (Figure 1G) revealed a decline in spatial learning in BDE (Day 4 latency = 14.33 ± 3.39 s) and VPA groups (Day 4 latency = 15.66 ± 3.41 s) versus CON (Day 4 latency = 8.26 ± 2.44 s vs. BDE, *p* = 0.0011; vs. VPA, *p* = 0.0002), mitigated by MT treatment (Day 4 latency = 10.59 ± 3.14 s vs. BDE, *p* = 0.0381). Probe trial metrics (Figure 1H) confirmed memory consolidation deficits in both BDE- and VPA-exposed rats, evidenced by reduced target quadrant occupancy (CON = 16.10 ± 2.88 s; BDE = 10.35 ± 2.54 s vs. CON, *p* = 0.0005; VPA = 10.09 ± 2.71 s vs. CON, *p* = 0.0002) and platform crossings (CON = 8 ± 2.33; BDE = 4.75 ± 1.91 vs. CON, *p* = 0.023; VPA = 4.38 ± 1.41 vs. CON, *p* = 0.002). MT treatment enhanced spatial recall performance (occupancy = 13.28 ± 3.03 s vs. BDE, *p* = 0.0238; crossings: 7.38 ± 2.33 vs. BDE, *p* = 0.042), approximated to the control.

### 3.2. Transcriptomics Profiles Indicate Both Mitochondrial and Synaptic Dysfunction Are Involved

**Figure 2:**
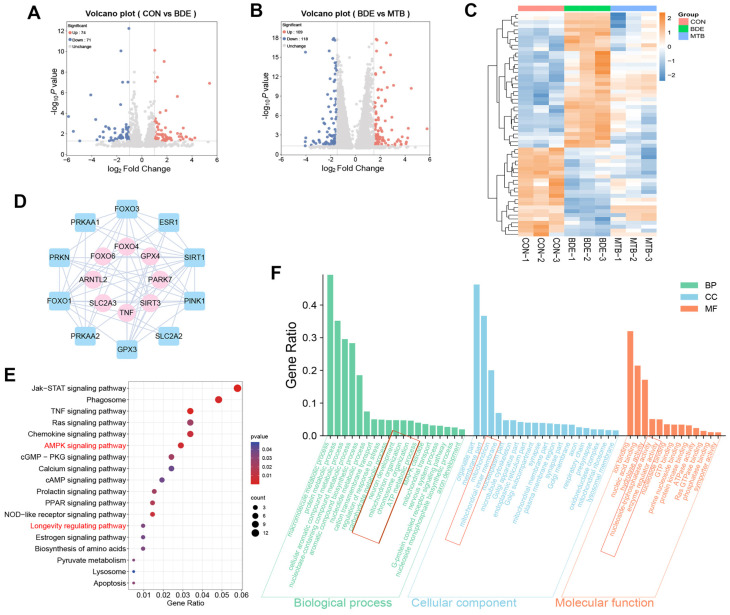
Transcriptomic profiles indicate that both mitochondrial and synaptic dysfunction are involved. (**A**,**B**) Differential expressed genes (DEGs, upregulated, downregulated, unaltered) with −log_10_ (*p* value) and log_2_ (fold change) axes, resulting from RNA-seq of hippocampus tissues in CON, BDE, and MTB groups (*n* = 3 rats). (**C**) Hierarchically clustered heatmap visualizing DEGs (log_2_FC > |1|, *p* < 0.05) expression pattern. (**D**) Cytoscape-generated protein–protein interaction (PPI) network presenting co-expression relationships between DEGs (pink) and those involved in SIRT1 signaling and mitochondrial function (blue). (**E**) KEGG pathway enrichment analysis of cross-group DEGs. (**F**) Gene Ontology (GO) enrichment landscape of tripartite comparisons, categorizing biological processes (BPs), molecular functions (MFs), and cellular components (CCs).

We performed high-throughput RNA sequencing to identify DEGs in the hippocampus of adolescent female rats after perinatal BDE-209-exposure and MT-pretreatment. As shown in Figure 2A,B, 145 genes were identified due to decaBDE exposure; 227 genes were differentially expressed under MT intervention, including 109 upregulated and 118 downregulated genes compared with the BDE group. Hierarchical clustering (Figure 2C) revealed that BDE-209 exposure specifically disturbed the expression of 58 transcripts, notably Tnf, Foxo4, Park7, and Sirt3, which were rescued by MT intervention. Mechanistic interrogation (Figure 2E) revealed the AMPK signaling pathway (*p* = 0.006) and longevity regulating pathway (*p* = 0.034) as the principal regulatory axis. Network modeling (Figure 2D) demonstrated that TNF regulated its co-expressed gene SLC2A2 (GLUT2, combined score = 0.403), whose translation was governed via deacetylation by SIRT1 (interaction score = 0.89). Concurrently, FOXO4 regulated ESR1 and FOXO3 (combined score = 0.459), PARK7 regulated PINK1 (combined score = 0.999), and SIRT3 regulated SIRT1 (combined score = 0.46), the latter being an effector of the AMPK/SIRT1 axis (interaction score = 0.733). Functional annotation (Figure 2F) maps these perturbations to mitochondrial organization (BP: *p* = 0.021) and neuron development (dendritic spine formation, BP: *p* = 0.016), mitochondria (CC: *p* = 0.027) and mitochondrial membrane (inner membrane, CC: *p* = 0.039), and ATPase activity (NAD+-dependent epigenetic regulation, MF: *p* = 0.024), revealing bioenergetic compromise.

### 3.3. Melatonin Improves Redox Homeostasis and Reduces Apoptosis of Hippocampal Nerve Cells After decaBDE Exposure

**Figure 3:**
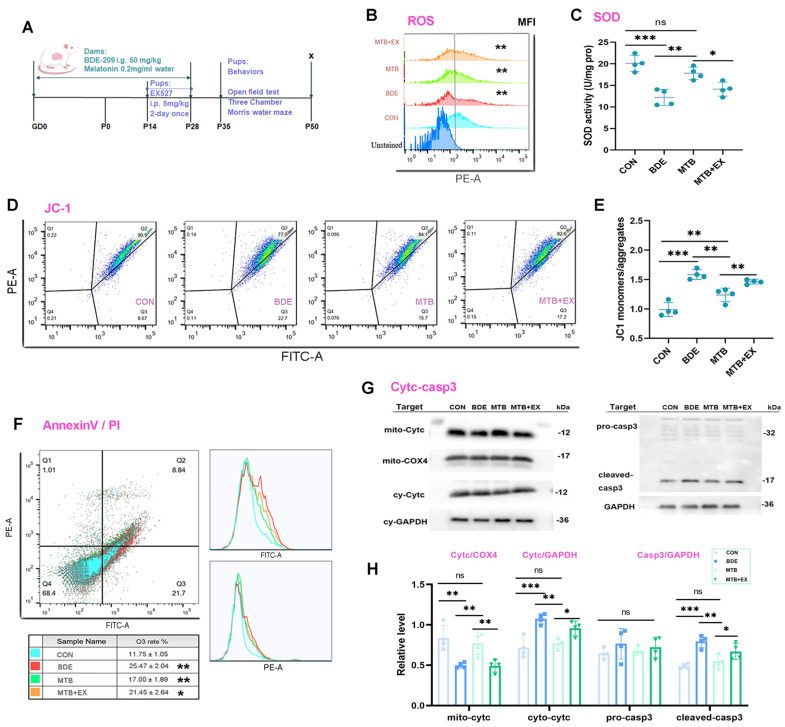
Melatonin improves redox homeostasis and reduces apoptosis of hippocampal nerve cells after decaBDE exposure. (**A**) Experimental design and schedule of dams and offspring rats; materials were from the Figdraw 2.0 platform under license number IOUAY4bb92. (**B**) Fluorescence intensity of cellular ROS marker DCF; MFI: mean fluorescence intensity. (**C**) SOD2 enzyme activity (U/mg pro). (**D**,**E**) Mitochondrial membrane potential marker JC1 monomers and aggregates. (**F**) Apoptosis rate (Q3) reflected as percentage of Annexin V-FITC (+)/PI (−) cells, with corresponding mean fluorescence intensity. (**G**,**H**) Representative Western blotting bands of proteins in mitochondrial apoptotic pathway and quantification normalized to GAPDH. Data are expressed as mean ± SD, *n* = 3~4, * *p* < 0.05, ** *p* < 0.01, *** *p* < 0.001; ns, non-significant.

Mitochondrial dysfunctions are associated with neurodevelopmental disorders, including autism and intellectual disability, which can be ameliorated by SIRT1 activation or elevation [37]. Based on molecular information from transcriptomics profiles, we further investigated whether mitochondrial homeostasis maintenance in the hippocampus under melatonin and deca-BDE administration occurred through the SIRT1–SIRT3 axis, utilizing inhibitor EX527 (Figure 3A). Oxidative stress was remarkably induced in BDE-209-exposed rats, as verified by reduction in SOD activity (60.7% of CON, *p* < 0.001) and increased ROS levels in hippocampal nerve cells (1.940-fold to CON, *p* < 0.001). Concomitantly, mitochondrial membrane potential (Δψm) disruption (JC1 monomers/aggregates: 1.583 ± 0.082, *p* < 0.001 vs. CON), release of cytochrome c from mitochondria (cyto-cytc/mito-cytc: 2.156 ± 0.176, vs. 0.873 ± 0.194, *p* < 0.001), and, finally, resulted apoptosis were observed after BDE-209 exposure, as shown by the increase in cleaved-caspase-3 (1.634-fold to CON, *p* < 0.001) and AnnexinV-FITC-positive cells (2.279-fold to CON, *p* < 0.001). Melatonin treatment restored redox-oxidative homeostasis (MTB/BDE: SOD activity, 1.458, *p* = 0.003; ROS-MFI, 74.2%, *p* = 0.001), Δψm (JC1 monomers/aggregates: 1.238 ± 0.116, *p* = 0.001 vs. BDE) and cytochrome c release (cyto-cytc/mito-cytc: 1.039 ± 0.273; MTB vs. BDE, *p* = 0.001), and subsequent caspase-3 activation (MTB/BDE: 68.5%, *p* = 0.003) along with apoptosis (MTB/BDE: 66.7%, *p* = 0.003), whereas EX527, which inhibited SIRT1 activity but not expression, blocked the abovementioned melatonin’s restoration on mitochondrial homeostasis (MTB-EX vs. MTB, *p* < 0.05, Figure 3B–E) and ultimately nerve cells apoptosis (MTB-EX vs. MTB, *p* < 0.05, Figure 3F–H).

### 3.4. Melatonin Maintains Mitochondrial Dynamics Through SIRT1 Activation in the Hippocampus of decaBDE-Exposed Rats

**Figure 4:**
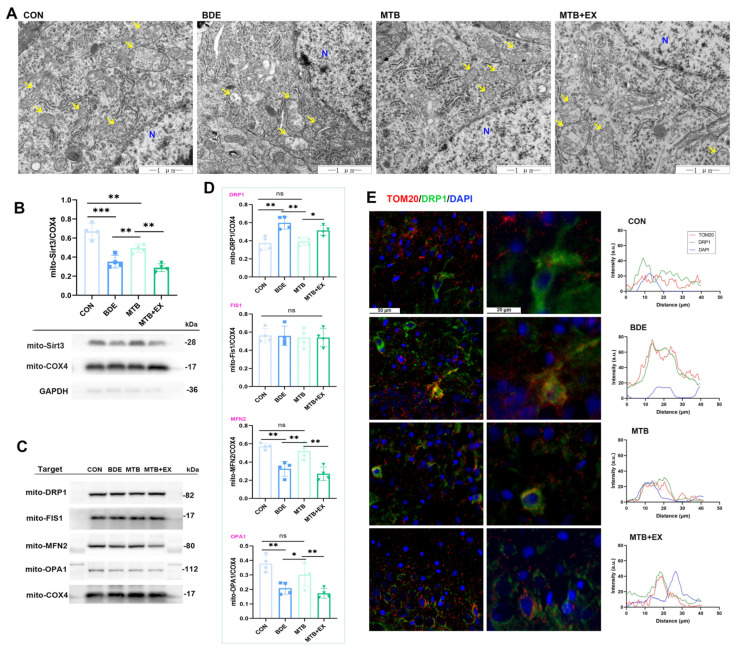
Melatonin maintains mitochondrial dynamics through Sirt1 activation in the hippocampus of decaBDE-exposed rats. (**A**) Ultrastructure of mitochondria (yellow arrow) in hippocampal neurons by TEM. N, nucleus; scale bar, 1 μm. (**B**) Mitochondrial Sirt3 protein level. (**C**,**D**) Mitochondrial fission and fusion proteins (DRP1, Fis1; MFN2, OPA1) expression, normalized to COX4 (*n* = 4). (**E**) Representative immunofluorescence staining of DRP1 (green) and co-localization analysis with TOM20 (red) within hippocampal neurons. Data are expressed as mean ± SD, * *p* < 0.05, ** *p* < 0.01, *** *p* < 0.001; ns, non-significant.

Mitochondrial dynamics disorder contributes to cognitive and motor impairment, consisting of coordinated shifts between fusion and fission, which are essential for maintaining mitochondrial morphology, quantity and position within neurons [38]. Here, TEM revealed that the inner membrane was folded to form obvious ridges and the outer membrane was intact in CON; on the contrary, abnormal mitochondrial ultrastructure, such as swollen and crista separation or disappearance in hippocampal neurons, caused by BDE-209 (BDE) were effectively attenuated by melatonin (MTB, Figure 4A). As a mitochondrial sirtuin, SIRT3 regulates mtROS production and mediates the deacetylation of antioxidant enzymes, thus protecting the integrity of mitochondria [39]. Two proteins pivotal for mitochondrial membrane fusion are mitofusin-2 (MFN2) and optic atrophy 1 (OPA1), whereas the recruitment of cytoplasmic dynamin-related protein 1 (DRP1) and its anchoring in mitochondria by mitochondrial fission protein 1 (FIS1) are required for fission [40]. We further explored these proteins involved in mitochondrial dynamics: SIRT3 in mitochondria was significantly reduced by BDE-209 exposure (52.5% of CON, *p* < 0.001, Figure 4B), as well as fusion protein MFN2 (57.2%/CON, F_(3,12)_ = 16.048, *p* < 0.001) and OPA1 (55.0%/CON, F_(3,12)_ = 11.158, *p* = 0.001), while mitochondrial DRP1 was increased (1.600-fold to CON, F_(3,12)_ = 11.511, *p* = 0.001), without significant alteration on the FIS1 level (F_(3,12)_ = 1.413, *p* = 0.287, Figure 4C,D). Compared with treatment with BDE alone, melatonin pretreatment reversed these changes in mitochondrial protein levels (e.g., mito-SIRT3: 1.402/BDE, *p* = 0.006) and the recruitment of DRP1, as verified by decreased colocalization of DRP1 and TOM20 through immunofluorescence (Figure 4E). These protective effects of melatonin against BDE-209-induced neuronal mitochondrial damage were impeded by EX527 supplementation.

### 3.5. Melatonin Promotes SIRT1-Dependent Mitochondrial Biogenesis in the Hippocampus of decaBDE-Exposed Rats

**Figure 5:**
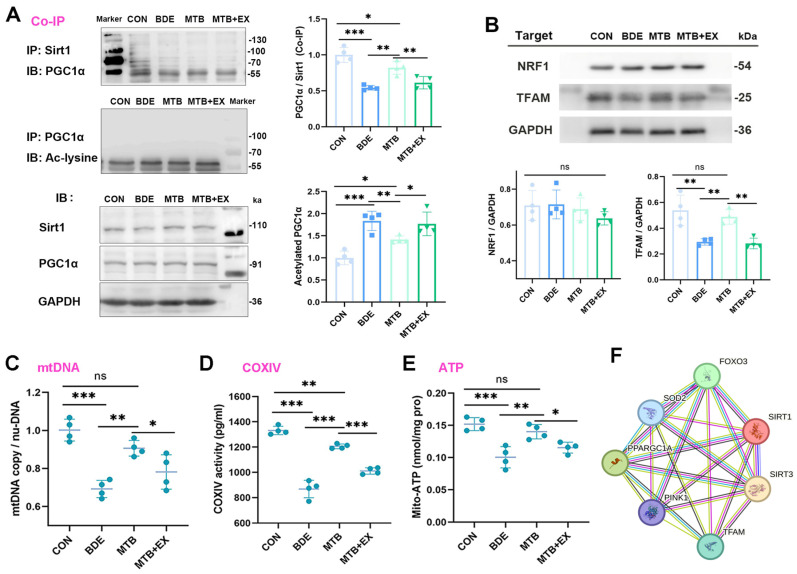
Melatonin promotes mitochondrial biogenesis via SIRT1-PGC1α-TFAM signaling. (**A**) Representative immunoblotting (IB) bands of Sirt1-immunoprecipitated PGC1α and acetylated-PGC1α. (**B**) Representative Western blotting bands of NRF1 and TFAM, quantification normalized to GAPDH (*n* = 4). (**C**) Mitochondrial DNA (mtDNA) copy number relative to nuclear DNA (nuDNA) quantified by qPCR. (**D**) Complex IV (cytochrome c oxidase (COX) IV) enzyme activity (pg/mL). (**E**) Mitochondrial ATP content of hippocampal neural cells (*n* = 3~4). Data are expressed as mean ± SD, * *p* < 0.05, ** *p* < 0.01, *** *p* < 0.001; ns, non-significant. (**F**) Proteins interaction network constructed using STRING to illustrate crosstalk between SIRT1 and SIRT3 in the regulation of mitochondria homeostasis.

The maintenance of neuronal homeostasis and function depends on proper mitochondrial dynamics, biogenesis, and mitophagy. The SIRT1-dependent PGC-1α/NRF1/TFAM pathway is pivotal for regulating mitochondrial biogenesis; especially, TFAM triggers the transcription and replication of mtDNA [41]. Therefore, we estimated whether mitochondrial biogenesis-regulating molecules were altered upon BDE-209 exposure. As shown by co-immunoprecipitation (Figure 5A), SIRT1-combined PGC-1α was reduced in the hippocampus of BDE-209-exposed rats (0.543 ± 0.032, *p* < 0.001 vs. CON); simultaneously, acetylated PGC-1α was elevated upon BDE-209 treatment (1.837 ± 0.217, *p* < 0.001 vs. CON). With respect to the downstream signal, TFAM protein expression, while not NRF1, was significantly decreased (F_(3,12)_ = 14.436, *p* < 0.001, Figure 5B). Consequently, BDE-209 administration induced significant decline in mitochondrial DNA (mtDNA) copy number (mtDNA/nuDNA, 0.692 ± 0.045, Figure 5C), as well as oxidative phosphorylation (OXPHOS) inhibition, indicated by reduction in complex IV (COXIV) activity (65.2% of CON, Figure 5D) and mitochondrial ATP levels (65.8% of CON, Figure 5E).

As expected, melatonin reversed the suppressed mitochondrial biogenesis induced by BDE-209, as manifested by increase in mtDNA contents (0.906 ± 0.041, *p* = 0.002 vs. BDE). In addition, COXIV activity (1.389-fold to BDE, *p* < 0.001) and mitochondrial ATP (1.402-fold to BDE, *p* = 0.002) were also elevated. Accordingly, we found that PGC-1α combined with SIRT1 was further promoted by melatonin (PGC-1α/SIRT1: 1.512-fold to BDE, *p* = 0.002), along with downregulated PGC-1α acetylation (ace-PGC-1α: 77.0% of BDE, *p* = 0.009) and augmented protein expression of TFAM (1.662-fold to BDE, *p* = 0.002). However, in response to EX527 co-treatment, the protective effects of melatonin on both mitochondrial biogenesis and molecular regulators were suppressed.

### 3.6. Melatonin Triggers SIRT3-FOXO3a-Targeted Gene Expression Regulated by SIRT1

**Figure 6:**
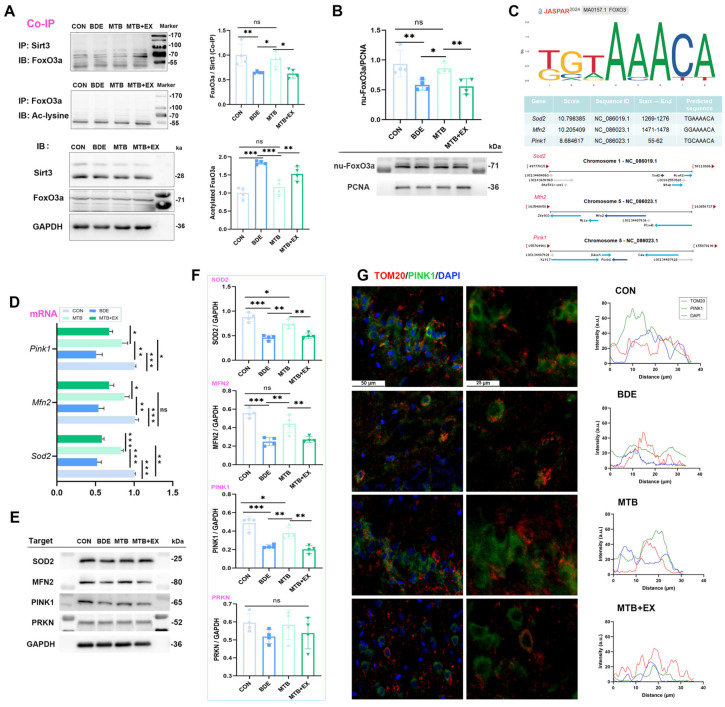
Melatonin triggers SIRT3-FoxO3a-targeted gene expression regulated by SIRT1. (**A**) Representative immunoblotting (IB) bands of Sirt3-immunoprecipitated FoxO3a and acetylated-FoxO3a. (**B**) Nuclear FoxO3a protein levels normalized to PCNA (*n* = 4). (**C**) Predicted transcription factor binding sites (TFBS) of genes (*Sod2*, *Mfn2*, *Pink1*) by JASPAR. (**D**) Transcription of FoxO3a-targeted genes detected by real-time PCR (*n* = 3~4). (**E**,**F**) Protein expression of SOD2, MFN2, and PINK1/PRKN quantification normalized to GAPDH (*n* = 4). (**G**) Immunofluorescence co-localization of PINK1 (green) with mito-marker TOM20 (red) within hippocampal neural cells. Data are expressed as mean ± SD, * *p* < 0.05, ** *p* < 0.01, *** *p* < 0.001; ns, non-significant.

As mentioned above, melatonin restored mitochondrial SIRT3 level in the hippocampus of BDE-209-exposed rats, and SIRT3 has been proven to promote mitophagy by activating the FOXO3a-PINK-Parkin signaling pathway [42]. We further detected FOXO3a acetylation using co-immunoprecipitation, and found that the deacetylation of FOXO3a by SIRT3 was correspondingly decreased by BDE-209 (0.651 ± 0.022, *p* = 0.005 vs. CON), whereas acetylated FoxO3a was increased (1.841 ± 0.053, *p* < 0.001 vs. CON, Figure 6A), which prevented its transcriptional activity in the nucleus (nu-FoxO3a: 62.2% of CON, Figure 6B). As FOXO regulates the expression of nuclear genes that mediate mitochondrial biogenesis, dynamics, and mitophagy [43], we predicted transcription factor binding sites (TFBS) of target genes with FOXO3 utilizing JASPAR. The predicted binding sequences with the highest scores for SOD2, MFN2, and PINK1 are presented in Figure 6C. Correspondingly, mRNA levels of these genes were downregulated by BDE-209 (*p* < 0.001 vs. CON, Figure 6D). Accordingly, protein expression of SOD2, MFN2, and PINK1, but not total PRKN (F_(3,12)_ = 1.136, *p* = 0.374), decreased in the hippocampus of rats upon BDE-209 exposure (*p* < 0.001 vs. CON, Figure 6E,F), Furthermore, ubiquitin-dependent mitophagy requires PINK1 and the recruitment of Parkin (PRKN) to mitochondria, whereas the co-localization of PINK1 with TOM20 in hippocampal neurons was elevated after BDE-209 exposure (Figure 6G), reflecting the failure of PINK1 import and, thus, the blockage of PRKN translocation upon mitochondrial depolarization.

Melatonin pretreatment improved FOXO3a deacetylation via SIRT3 (1.419-fold to BDE, *p* = 0.020), promoted FOXO3a nuclear retention (1.48-fold to BDE, *p* = 0.016), and enhanced the expression of SOD2, MFN2, and PINK1 (*p* < 0.01, MTB vs. BDE), which jointly facilitated mitophagy and mitochondrial homeostasis. However, these restorations were abrogated under SIRT1 pharmacological inhibitor EX527, and their protein–protein interaction (PPI) networks were constructed by STRING, as shown in Figure 5F, indicating the role of the SIRT1–SIRT3 axis upon both neurotoxicity of BDE-209 and neuroprotection of melatonin.

### 3.7. Restoration of Melatonin in Hippocampal Synaptic Function Is Abolished by SIRT1 Inhibitor

**Figure 7:**
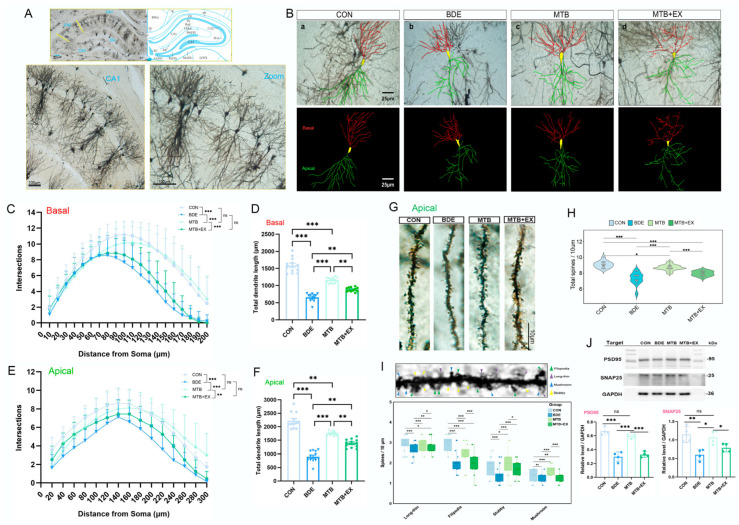
Restoration of melatonin in hippocampal synaptic function is abolished by SIRT1 inhibitor. (**A**) Representative Golgi-stained hippocampal sections (CA1, Cornu Ammonis 1); lower right is an enlarged version of the left (scale bar, 100 μm). (**B**) Reconstructions of the dendrites of CA1 pyramidal neurons (scale bar, 25 μm). (**C**,**E**) Intersections of basal and apical dendrites assessed by Sholl analysis. (**D**,**F**) Total length (μm) of basal and apical dendrites assessed by ImageJ. (**G**,**H**) Apical dendritic spine density of CA1 pyramidal neurons. (**I**) Representative diagram of morphological classification for Golgi spines (filopodia, long-thin, stubby, mushroom) and spine density in each type (*n* = 12 neurons from 3 rats per group). (**J**) Representative Western blotting bands of synaptic proteins and quantification normalized to GAPDH (*n* = 4). Data are expressed as mean ± SD, * *p* < 0.05, ** *p* < 0.01, *** *p* < 0.001; ns, non-significant.

Behavioral deficits have been postulated to result from abnormalities in dendritic structure and consequently disruption of neuronal circuits [44]. Here, the dendritic arborization and spine density of pyramidal neurons in the hippocampal CA1 region were determined by Golgi–Cox staining (Figure 7A). As shown in Figure 7B–F, branch points of both basal and apical dendrites were declined in BDE-209-exposed rats (BDE vs. CON, *p* < 0.001), and dendritic length also exhibited marked reduction compared with control (basal/μm: 658.8 ± 122.0 vs. 1589 ± 63.50; apical/μm: 900.7 ± 61.00 vs. 2218 ± 84.65; *p* < 0.0001). MT intervention reversed above dendritic changes observed in BDE group (branches: MTB vs. BDE, *p* < 0.001; apical dendrite length: 1753 ± 78.15, *p* < 0.0001), which was abrogated by SIRT1 inhibitor EX527 (MTB + EX: 1387 ± 153.1, *p* < 0.001). Moreover, assessment on apical spines (Figure 7G–I) indicated that total spine density (BDE = 7.31 ± 0.79 vs. CON = 9.10 ± 0.44/10 μm, *p* < 0.001) and mature spine density (mushroom-shaped + stubby-shaped: 2.02 ± 0.41 vs. 3.07 ± 0.49/10 μm, *p* < 0.0001) were significantly lower than the control. MT treatment partially restored complexity metrics (Filopodia: MTB = 2.48 ± 0.46/10 μm; mature spines: MTB = 3.17 ± 0.33/10 μm; *p* < 0.0001 vs. BDE), whereas combining EX527 with MT elicited repressive effects, and reduced densities to near BDE-equivalent levels (Filopodia: MTB + EX = 2.03 ± 0.38/10 μm vs. MTB, *p* = 3.81 × 10^−7^; Stubby: MTB + EX = 1.74 ± 0.39/10 μm vs. MTB, *p* = 0.0104).

Furthermore, the expression of both presynaptic SNAP25 (BDE = 0.603 ± 0.073 vs. CON = 1.146 ± 0.109, *p* = 0.0033) and postsynaptic PSD95 (BDE = 0.300 ± 0.068 vs. CON = 0.667 ± 0.054, *p* < 0.001) was decreased by BDE-209 exposure, which was restored by MT administration (MTB: SNAP25, 1.068 ± 0.091, *p* = 0.0102; PSD95, 0.592 ± 0.031; *p* < 0.001), while also abolished by SIRT1 activity inhibitor EX527 (Figure 7J). Taken together, these findings suggest that MT improved dendritic spine abnormalities and synaptic connections in the hippocampi of BDE-209-treated rats, partially through SIRT1 activation.

## 4. Discussion

Epidemiological studies have indicated that exposure to PBDEs during early life causes neurobehavioral deficits, including poor attention, low intelligence, social difficulty, and hyperactivity/impulsivity [45,46]. Here, we explored the involvement of mitochondrial quality control and its regulation by the SIRT1–SIRT3 axis in decaBDE-induced neurodevelopmental disorders. We found that perinatal BDE-209 exposure caused autism-relevant behavioral changes in female offspring rats, which were attenuated by melatonin supplementation, and the molecular mechanisms identified through RNA sequencing revealed that AMPK and longevity signaling were primarily involved.

Children prenatally exposed to VPA have an increased opportunity of incidence of autism [47]. Analogously, VPA model rats presented ASD-like phenotypes including decreased preference for social novelty, increased marble burying behavior, and learning impairment [48]. Consistent with a prior report that perinatal exposure to 50 mg/kg BDE-47 caused mild autistic-like behaviors in offspring, which were less severe than those observed in pups maternally exposed to VPA [46], BDE-209 exposure throughout gestation and lactation also reduced sociability and social novel preference in adolescent female rats, together with anxiety and poor spatial learning and memory acquisition, whereas total exploration in open-field test differed between VPA models and BDE-209-exposed rats, indicating that externalizing problems such as hyperactivity are signs of disturbed neurodevelopment after BDE-209 exposure [49].

Importantly, melatonin, a hormone secreted rhythmically by the pineal gland, mitigated BDE-47-induced neurotoxicity by preventing neuronal apoptosis and loss through the restoration of mitophagy activity and mitochondrial function [50]. Furthermore, melatonin supplementation during late gestation and early postnatal development rescued the social deficits of the VPA model [27]. In this study, exogenous maternal melatonin ameliorated BDE-209-induced behavioral impairments, manifested as improvements in social performance, key phenotypes of autism, anxiety, and cognitive ability. Mechanistically, after RNA sequencing, we explored the potential molecular network in the hippocampus and revealed that decaBDE-specific transcriptomic disturbance, notably across *Tnf*, *Foxo4*, *Park7*, and *Sirt3*, was restored after melatonin intervention, indicating a principal role for AMPK signaling and longevity-regulating pathways in mitochondrial organization and neuron development.

Mitochondrial dysfunctions and metabolic disturbances are more common in individuals with ASD than in the general population, and biomarkers such as lactate, ATP, and CoQ10, as well as mtDNA variants and copy number, are associated with clinical features [51]. Perinatal PBDE exposure induces mitochondrial dysfunction in conjunction with disordered antioxidant response, and, thus, increases child’s susceptibility to autism [52]. Increased ROS production, decreased SOD activity, and apoptosis via the mitochondrial pathway were observed in the hippocampus of BDE-209-treated rats; unsurprisingly, ultrastructural morphological changes in mitochondria, such as swollen and crista separation, occurred. Moreover, treatment with melatonin restrained BDE-209-induced morphological and functional impairments and redressed redox homeostasis.

Mitochondrial dynamics not only regulate the morphology but also facilitate content exchange and maintenance of mtDNA and OXPHOS activity, thereby keeping mitochondrial integrity [12]. The mtDNA copy number is correlated with mitochondria amount, whereas OXPHOS, a mitochondrial metabolic pathway involving Complexes I to V, generates ATP efficiently [13]. Here, molecular alterations in the dynamic processes of fusion/fission focused on mitochondrial DRP1 assembly and reduction in MFN2 expression. Moreover, decreased mtDNA copies, complex IV activity, and mitochondrial ATP content were concurrently discovered in the rat hippocampus after BDE-209 exposure, in accordance with recent findings upon neonatal BDE-47 exposure (PND 10 to 16) [53]. In contrast, enhancing mitochondrial fusion through adenovirus-mediated Mfn2 overexpression was verified to rescue BDE-47-induced mitochondrial dynamics, morphological impairments, and the resulting neuronal apoptosis [54], whereas neither stimulation of mitochondrial fission by Fis1 overexpression nor suppression by mitochondrial division inhibitor-1 (Mdivi-1) could reverse these damages, further demonstrating the pivotal role of MFN2.

Further integrating mitochondrial biogenesis and mitophagy, consistent with previous studies [26,55], suppressed SIRT1-dependent deacetylation of PGC1α and resultant TFAM expression were observed in adolescent rats’ hippocampi after BDE-209 exposure, illuminating the above alterations in mtDNA and ATP generation. Moreover, reduced mitochondrial SIRT3 and FOXO3a transcriptional activity were correspondingly presented as downstream of SIRT1 [56]. Melatonin promoted the accumulation of mitochondrial SIRT3 and the resulting deacetylation and nuclear retention of FOXO3a. As a transcription factor, genes downstream of FOXO3a, including *Sod2*, *Mfn2*, and *Pink1*, were accordingly altered upon BDE-209 and melatonin treatment. Indeed, PINK1/Parkin-mediated mitophagy was suppressed by BDE-47 in striatal neurons of rats, while restored by melatonin [50]. Moreover, SIRT3 reversed mitophagy inhibition by activating the FOXO3a-PINK1-Parkin signaling pathway [42]. Here, we found that destabilized PINK1 on the outer mitochondrial membrane (OMM) was induced by BDE-209, which was reversed by melatonin. However, these protective effects were abolished by the selective blocker upon SIRT1 activity, EX527. Given that melatonin can relieve BDE47-induced apoptosis and mitochondrial dysfunction through the AMPK-SIRT1-PGC1α pathway [57] concurrently, and SIRT1 alleviated neuronal mitochondrial bioenergetics deficits by activating SIRT3 [58], we speculated that the SIRT1–SIRT3 axis exhibited an essential role in both BDE-209 neurotoxicity and melatonin intervention. Moreover, melatonin triggered SOD2 deacetylation independent of SIRT1, which was blocked by the SIRT3-selective inhibitor 3-TYP [59]; therefore, SOD2 activity enhancement induced by melatonin here is likely attributed to SIRT1–SIRT3-FOXO3a regulation.

Alterations in the morphology and plasticity of dendritic spines are common hallmarks of neurodevelopmental disorders [44], and also have been involved in behavioral impairments induced by perinatal BDE-47 and/or VPA exposure [46]. Synaptosome associated protein 25 (SNAP-25), a presynaptic member regulating neurotransmitters release, could facilitate PINK1-mediated mitophagy; mechanistically, SNAP25 depletion destabilized PINK1 and blocked Parkin translocation to the mitochondria [60]. Intriguingly, suppression of PINK1-mediated mitophagy and SNAP25 downregulation were observed in the hippocampi of juvenile rats after neonatal BDE-47 and BDE-209 exposure, respectively [50,61]. Conversely, melatonin reversed BDE-209-induced synaptic dysfunction and upregulated the expression of SNAP-25 and postsynaptic density-95 (PSD-95), concurrently restoring dendritic spine morphology and density. Considering the role of SIRT1 in improving cognition and as an underlying antidepressant, majorly focused on hippocampal synaptic plasticity (dendritic spines, PSD95, and synaptophysin) and neurogenesis (GAP43, MAP2, and activity-regulated cytoskeletal-associated protein (Arc)) [62,63], inhibition of SIRT1 also abolished these melatonin effects contributing to autism-relevant behavioral amelioration. More recently, the NAD+/SIRT3 axis has been identified as a promising therapeutic target for addressing cognitive dysfunctions [64]; as behavioral alterations caused by BDE-209 are complicated, and there are pleiotropic implications of melatonin’s impact on sirtuin, SIRT3-dependent regulation needs further validation in subsequent investigation through specific inhibition or knockdown.

As the estimated prevalence of ASD is higher in males than in females (3.7% in boys and 1.5% in girls) [2], future work should include male cohorts to generalize the findings; the biological sex-specific etiological mechanisms for autism should be inquired thoroughly, like androgen receptor (AR), estrogen receptor alpha (ESR1), and retinoic acid-related orphan receptor-alpha (RORA), especially upon the exposure of endocrine-disrupting chemicals. Given that melatonin secretion pattern was aberrant in individuals with ASD, particularly decreased at night and with altered circadian rhythms, melatonin treatment near the dark phase improved social behavioral deficits and cognition [65]. Therefore, a more appropriate period of administration should be considered.

## 5. Conclusions

Melatonin attenuated perinatal BDE-209 exposure-induced autism-relevant behaviors, and the underlying mechanism majorly implicated SIRT1-dependent modulation in mitochondrial fission/fusion dynamic remodeling, PGC1α/TFAM-determined biogenesis, SIRT3-FOXO3a transcriptional activity, and hippocampal synaptic plasticity. By integrating transcriptomics profiles, this study highlighted the SIRT1–SIRT3 axis in mitochondrial quality control and behavioral effects, providing novel intervention for PBDEs’ neurodevelopmental impairment.

## Figures and Tables

**Table 1:** Primer sequence used for quantitative real-time PCR.

Gene	Accession No.	Prod bp	Primer Sequences (5′-3′)
*Sod2*	NM_017051	130	Forward: -TCCCTGACCTGCCTTACGACTATG- Reverse: -TCGTGGTACTTCTCCTCGGTGAC-
*Mfn2*	NM_001429969	84	Forward: -TCCACAGCCATTGCCAGTTCAC- Reverse: -CCGCACAGACACAGGAAGAAGG-
*Pink1*	NM_001438119	105	Forward: -GAGGAGAAGCAGGCGGAGAG- Reverse: -TCGTGTGTCCAGTGGGTCAG-
*Gapdh*	NM_017008	143	Forward: -GGCACAGTCAAGGCTGAGAATG- Reverse: -ATGGTGGTGAAGACGCCAGTA-
*Mt-co1*	NC_001665.2	125	Forward: -GCCAGTATTAGCAGCAGGTATCA- Reverse: -GGTGGCCGAAGAATCAGAATAG-
*Ndufv1*	NC_051336.1	89	Forward: -GACCGAGTCTGAGGATGATATGC- Reverse: -AGTACCTGCGGAGCCATTGT-

## Data Availability

The RNA data presented in the study are openly available in NCBI SRA at BioProject PRJNA143025.

## Abbreviations

The following abbreviations are used in this manuscript:

ASD	Autism spectrum disorder
PBDEs	Polybrominated diphenyl ethers
BFRs	Brominated flame retardants
DecaBDE/BDE-209	Decabromodiphenyl ether
DBDPE	Decabromodiphenyl ethane
OXPHOS	Oxidative phosphorylation
ATP	Adenosine triphosphate
mtDNA	Mitochondrial DNA
MQC	Mitochondrial quality control
SIRTs	Sirtuins
NAD	Nicotinamide adenine dinucleotide
PGC-1α	Peroxisome proliferator-activated receptor (PPAR)-γ coactivator-1α
TFAM	Mitochondrial transcription factor A
FOXO3a	Forkhead box O3
MFN2	Mitofusin 2
DRP1	Dynamin-related protein 1
PINK1	PTEN-induced putative kinase1
MMP	Mitochondrial membrane potential
VPA	Sodium valproate
PND	Postnatal day
OFT	Open-field test
MWM	Morris water maze
TEM	Transmission electron microscopy
SOD	Superoxide dismutase
ROS	Reactive oxygen species
DEGs	Differentially expressed genes
GO	Gene Ontology
KEGG	Kyoto Encyclopedia of Genes and Genomes
PPI	Protein–protein interaction
qPCR	Quantitative real-time PCR
Co-IP	Co-immunoprecipitation
FIS1	Mitochondrial fission protein 1
OPA1	Optic Atrophy 1
SNAP25	Synaptosome associated protein 25
PSD95	Postsynaptic density 95
Arc	Activity-regulated cytoskeletal-associated protein
